# Early Mortality in Patients with Biopsy-Only Glioblastoma: Clinical, Radiological and Molecular Predictors from a Retrospective Cohort

**DOI:** 10.3390/jcm15197639

**Published:** 2026-10-02

**Authors:** Massimiliano Domenico Rizzaro, Giulio Bertani, Claudia Fanizzi, Luigi Gianmaria Remore, Luigi Schisano, Stefano Borsa, Giorgio Fiore, Guido Del Vecchio, Jacopo Ascani, Elena Scagliotti, Manuela Caroli, Marco Locatelli

**Affiliations:** 1 Department of Pathophysiology and Transplantation, University of Milan, 20122 Milan, Italy; massimiliano.rizzaro@unimi.it (M.D.R.); luigi.remore@unimi.it (L.G.R.); jacopo.ascani@unimi.it (J.A.); marco.locatelli@unimi.it (M.L.); 2 Neurosurgery Unit, Fondazione IRCCS Ca’ Granda Ospedale Maggiore Policlinico, 20122 Milan, Italy; claudia.fanizzi@policlinico.mi.it (C.F.); luigi.schisano@policlinico.mi.it (L.S.); stefano.borsa@policlinico.mi.it (S.B.); giorgio.fiore@policlinico.mi.it (G.F.); elena.scagliotti@policlinico.mi.it (E.S.); manuela.caroli@policlinico.mi.it (M.C.); 3 Neuroradiology Unit, Fondazione IRCCS Ca’ Granda Ospedale Maggiore Policlinico, 20122 Milan, Italy; guido.delvecchio@policlinico.mi.it

**Keywords:** glioblastoma, biopsy, early mortality, prognostic factors

## Abstract

**Background/Objectives**: Patients with unresectable glioblastoma (GBM) undergoing biopsy alone represent a clinically heterogeneous population with poor survival and limited access to standard oncological treatment. We investigated clinical, radiological, and histomolecular factors associated with early mortality after biopsy. **Methods**: We retrospectively analyzed 93 consecutive cases of patients with IDH-wildtype GBM who underwent biopsy without tumor resection between 2013 and 2025. Early death was defined as death within three months of biopsy. Baseline clinical, radiological, and molecular variables were evaluated using univariable analyses and exploratory multivariable logistic regression. **Results**: Median overall survival was 3 months, and 48/89 patients with available survival data (53.9%) died within three months. Preoperative Karnofsky Performance Status (KPS) and MGMT promoter methylation status (available for 71/93 patients) were nominally associated with early mortality in univariable analyses, although these associations did not remain significant after FDR correction. Age was not significant in univariable analysis but was associated with early mortality after adjustment in the exploratory multivariable model based on 62 complete cases. Conventional radiological parameters showed no consistent association with early death. Exploratory analysis suggested a high-risk phenotype combining KPS ≤ 60 and FLAIR signal crossing the midline. Overall, 23/93 patients (24.7%) did not initiate any adjuvant treatment, while only 17/93 (18.3%) completed the Stupp regimen. **Conclusions**: Early mortality is frequent in biopsy-only GBM. Clinical and molecular factors may help identify particularly vulnerable patients at diagnosis, supporting individualized treatment planning and shared decision-making. External validation in larger multicenter cohorts is warranted.

## 1. Introduction

Glioblastoma (GBM) is the most common primary malignant brain tumor in adults, accounting for approximately 14% of all primary brain tumors and over 50% of all malignant primary brain tumors. Population-based studies have reported an annual incidence of approximately 3.5 cases per 100,000 population in Europe and 3.3 cases per 100,000 population in the United States [1,2]. Nearly two decades after its introduction, the Stupp protocol remains the treatment associated with the greatest survival benefit. Nevertheless, the prognosis of GBM remains poor, with a median overall survival of approximately 15 months despite optimal multimodal therapy [1,3].

A substantial proportion of patients with newly diagnosed GBM (estimated at 20–35%) are not candidates for surgical resection and undergo diagnostic biopsy alone [4,5]. Currently, no universally accepted definition of unresectable glioblastoma exists. According to the European Association of Neuro-Oncology (EANO), stereotactic biopsy should be performed when microsurgical resection is not considered safely feasible [6]. In routine clinical practice, unresectability is determined by multidisciplinary assessment based on tumor location, infiltrative growth, and the anticipated risk of postoperative neurological morbidity. The prognosis of patients with biopsy-only GBM is particularly poor and highly heterogeneous. Compared with patients eligible for resection, these individuals frequently present with poorer functional status, greater clinical frailty, and tumors involving deep or eloquent brain regions, all of which may adversely affect outcome. Previous studies have reported a median overall survival ranging from approximately 4 to 7.6 months in biopsy-only GBM cohorts [5,7,8,9,10].

In resectable GBM, age, preoperative Karnofsky Performance Status (KPS), MGMT promoter methylation status, and extent of resection are well-established prognostic factors associated with improved survival [11,12]. In particular, MGMT promoter methylation reduces expression of the MGMT DNA-repair protein, limiting the repair of temozolomide-induced DNA damage and thereby increasing tumor sensitivity to treatment [13]. By contrast, evidence regarding unresectable GBM remains limited and the available studies have reported heterogeneous findings. Among the variables most consistently associated with survival are age, functional status, MGMT promoter methylation, and the ability to receive oncological treatment [7,8,9,10]. Conversely, the prognostic role of radiological features, including tumor location and volume, remains controversial. Even among patients with resectable GBM, a substantial proportion do not complete the full Stupp regimen, with treatment-completion rates varying considerably across clinical series [14,15,16]. In contrast, the proportion of patients who are unable to initiate or complete standard oncological treatment has not been clearly established in biopsy-only GBM. This knowledge gap is clinically relevant because patients who die within three months of diagnosis often deteriorate before deriving any meaningful benefit from standard oncological treatment. Furthermore, evidence specifically addressing very early mortality in this population remains scarce.

The present study aimed to identify clinical, radiological, and histomolecular factors associated with death within three months of diagnosis in patients with biopsy-only glioblastoma. Early identification of patients at extremely high risk of early mortality may improve prognostic stratification, facilitate more informed treatment planning, and support shared decision-making at the time of diagnosis.

## 2. Materials and Methods

### 2.1. Study Population

We retrospectively reviewed patients treated at Fondazione IRCCS Ca’ Granda Ospedale Maggiore Policlinico, Milan, Italy, between 2013 and 2025. All tumors were retrospectively classified according to the 2021 WHO classification. IDH status was assessed by IDH1 R132H immunohistochemistry and, when required, by molecular analysis of IDH1/2. Tumors harboring IDH1/2 mutations, including cases historically classified as IDH-mutant glioblastoma, were excluded. For cases diagnosed before 2021, the original histopathological and molecular reports were retrospectively reviewed and diagnoses were reassigned according to current WHO criteria when sufficient information was available. IDH-wildtype diffuse astrocytic tumors lacking conventional histological features of glioblastoma were classified as molecularly defined glioblastomas when a WHO 2021 molecular criterion (TERT promoter alteration, EGFR amplification, or combined chromosome 7 gain/chromosome 10 loss) was documented. TERT promoter hotspot alterations were assessed using the MassARRAY Analyzer 4 system (Agena Bioscience, San Diego, CA, USA), based on MALDI-TOF mass spectrometry, while EGFR amplification was assessed by next-generation sequencing (NGS).

The decision to perform biopsy rather than surgical resection was made on an individual basis following multidisciplinary evaluation. Factors contributing to biopsy-only management included deep tumor location, multicentric disease, poor preoperative functional status, multiple pre-existing comorbidities, highly invasive radiological presentation, and advanced age. These factors were not mutually exclusive, and no single criterion was considered mandatory for biopsy-only management. Radiological variables were defined as follows: FLAIR crossing the midline indicated continuous FLAIR hyperintensity extending across the midline, while a contrast-enhancing butterfly pattern indicated continuous T1 contrast enhancement across the midline. Multicentric disease was defined by the presence of spatially separate tumor foci without radiological continuity. In multicentric tumors, primary anatomical location referred to the dominant lesion, based on lesion size or accessibility for biopsy, from which tissue sampling was performed. No distinction was made according to the biopsy technique employed, and both frame-based stereotactic, frameless stereotactic, and open (non-stereotactic) biopsy procedures were included in the analysis.

### 2.2. Postoperative Management

Patients who maintained an adequate postoperative performance status, as judged by the treating physician, were considered eligible for adjuvant treatment with temozolomide-based chemotherapy, with or without concomitant radiotherapy. The indication for radiotherapy, radiation dose, and chemotherapy regimen were determined at the discretion of the treating physician according to the patient’s overall clinical condition, performance status, and laboratory findings. Postoperative complications were defined as adverse events occurring within 2 weeks after biopsy. Emergency department (ED) visits were recorded throughout the entire available survival period, from biopsy to death or last follow-up. Disease-, procedure-, or treatment-related visits were identified by review of the corresponding clinical records.

### 2.3. Study Endpoints

The primary endpoint of the study was early death, defined as death occurring within 3 months after brain biopsy. This time point was prespecified as a pragmatic short-term mortality endpoint to identify a clinically vulnerable subgroup in whom therapeutic decision-making is particularly challenging. Secondary endpoints included overall survival (OS), calculated from the date of biopsy to death from any cause or last available follow-up, and the identification of baseline clinical, radiological, histopathological, and molecular factors associated with early death. In addition, an exploratory analysis evaluated whether predefined combinations of baseline variables (clinical-radiological phenotypes) could identify patient subgroups at particularly high risk of death within the first 3 months after biopsy.

### 2.4. Tumor Volume Calculation

By using the BrainLab software 2.0 (BrainLab AG, Kapellenstrasse, Feldkirchen, Germany), FLAIR and T1-ce tumor volume were automatically segmented and measured. Segmentation was reviewed by two expert users (L.G.R. and M.D.R.) and manually refined if deemed necessary. The calculated volume was expressed as a continuous variable.

### 2.5. Statistical Analyses

Overall survival (OS) was calculated from the date of biopsy to death or last follow-up, with surviving patients censored. Median OS and 95% confidence intervals (CIs) were estimated using the Kaplan–Meier method. The primary endpoint was early death, defined as death within 3 months after biopsy. The distribution of continuous variables was assessed using the Shapiro–Wilk test. As the main continuous variables showed significant departures from normality, they were summarized using medians and interquartile ranges (IQRs). Continuous variables were compared between patients with and without early death using the two-sided Mann–Whitney U test, whereas categorical variables were analyzed using the two-sided Fisher exact test. Frailty was assessed using the 5-item modified Frailty Index (mFI-5) [17], which was analyzed both as a continuous variable and, exploratively, using a predefined cut-off of ≥2 to identify patients with greater frailty. The association between mFI-5 and early death was evaluated using the same univariable framework applied to the other baseline clinical variables. Relative dose intensity was calculated as the ratio between the delivered and planned temozolomide dose intensity, expressed as a percentage.

Continuous radiological variables were additionally evaluated by univariable binary logistic regression and expressed as ORs per clinically relevant increments. Survival curves were compared using the Kaplan–Meier method and log-rank test. Benjamini–Hochberg false-discovery-rate correction was applied across all exploratory univariable categorical comparisons. A restricted exploratory analysis of predefined two-factor clinical-radiological phenotypes was performed. To minimize overfitting, only prespecified two-variable combinations were evaluated. Early-death rates were reported with Wilson 95% CIs and compared using Fisher’s exact test. An exploratory multivariable binary logistic regression model was fitted to assess independent predictors of early death. Four clinically relevant covariates were selected a priori: age (per 10-year increase), preoperative Karnofsky Performance Status (per 10-point decrease), MGMT promoter methylation status, and FLAIR signal crossing the midline. These variables were selected on their clinical relevance and their potential prognostic association with early mortality. The incremental prognostic contribution of mFI-5 was additionally explored by adding the score to the original multivariable model in a sensitivity analysis. Complete-case analysis was performed for the multivariable model. Missingness for each model covariate was reported separately, and a sensitivity analysis compared baseline characteristics and outcome between patients included and excluded from the complete-case analysis. Adjusted ORs with 95% CIs were reported, and model performance was assessed using the likelihood-ratio test and area under the receiver-operating-characteristic curve (AUC). All statistical analyses, Kaplan–Meier survival curve and AUC generation were performed using SPSS software (version 27.0; IBM Corp., Armonk, NY, USA).

### 2.6. Ethics Statement

The study was conducted in accordance with the Declaration of Helsinki and was approved by the Ethics Committee of the enrolling institution according to national regulations (IRB#275/2013, approved on February 2013). Given the retrospective nature of the study, the requirement for written informed consent was waived.

## 3. Results

### 3.1. Patient Characteristics

**Figure 1:**
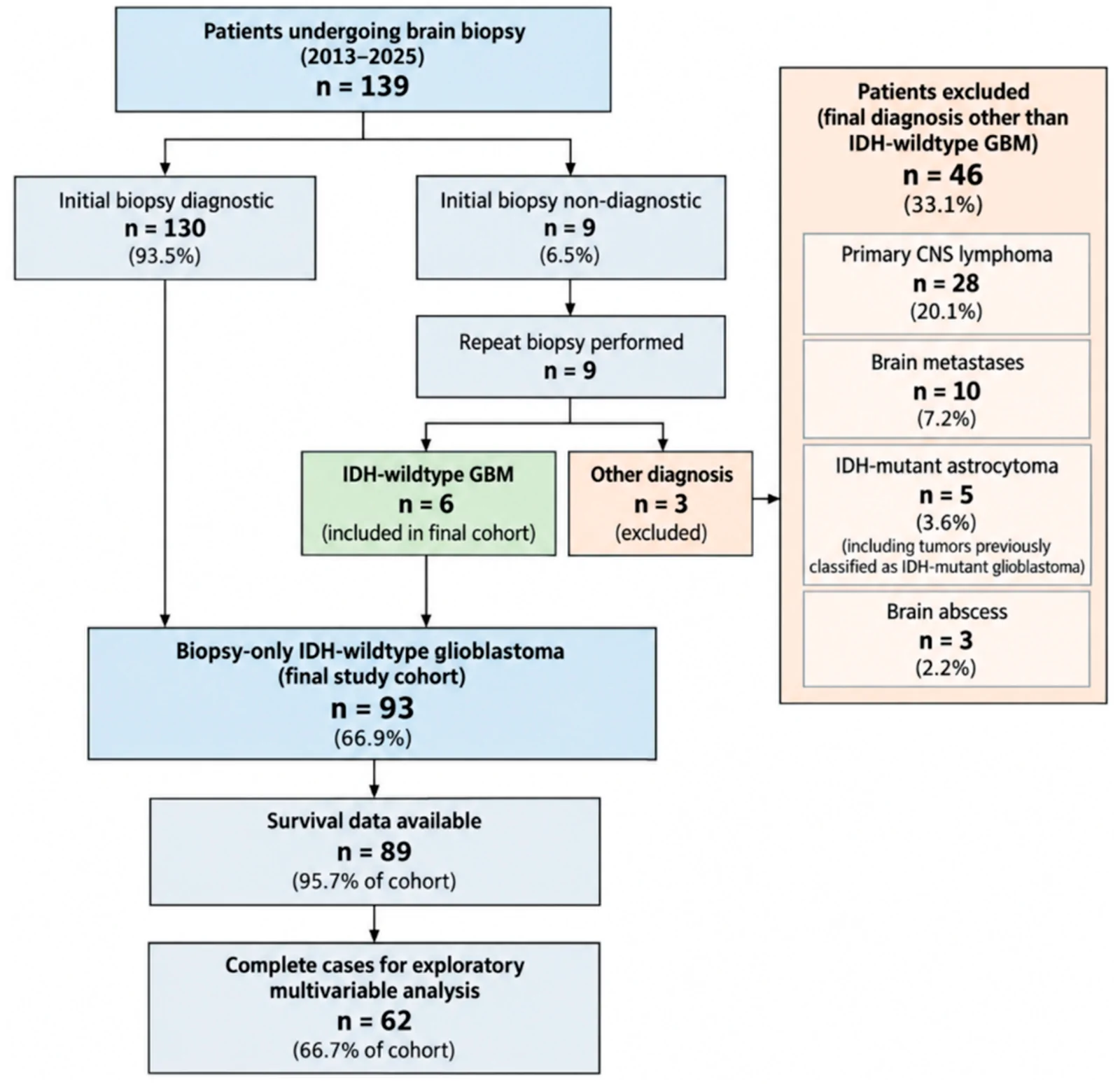
Flow diagram of patient selection and study cohort definition. GBM, glioblastoma; CNS, central nervous system; IDH, isocitrate dehydrogenase.

A total of 139 patients undergoing stereotactic biopsy between 2013 and 2025 were screened. Following the diagnostic work-up, 46 patients with diagnoses other than IDH-wildtype glioblastoma were excluded, resulting in a final cohort of 93 patients. Nine initial biopsies were non-diagnostic and underwent repeat biopsy; six subsequently yielded a diagnosis of IDH-wildtype glioblastoma and were included in the final cohort. The patient selection process is summarized in Figure 1.

The study cohort comprised 93 patients, including 47 women (50.5%) and 46 men (49.5%). Median age at biopsy was 70 years (IQR, 61–77), with a minimum age of 46. Median preoperative KPS was 80 (IQR, 70–80). Median immediate postoperative KPS was 80 (IQR, 70–80). The median mFI-5 score was 1 (IQR, 1–2; range, 0–4), and 30 patients (32.3%) had an mFI-5 score ≥ 2. Factors contributing to biopsy-only management were frequently overlapping. Deep tumor location was present in 54/93 patients (58.1%), multicentric disease in 58/93 (62.4%), mFI-5 ≥ 2 in 30/93 (32.3%), a contrast-enhancing butterfly pattern in 27/93 (29.0%), preoperative KPS ≤ 60 in 16/93 (17.2%), and age ≥ 80 years in 15/93 (16.1%). Within 2 weeks after biopsy, postoperative complications were documented in 8 of 93 evaluable patients (8.6%). Among the postoperative complications, intracranial hemorrhage was the most frequent event (5/8), followed by newly developed neurological deficits (1/8), seizures (1/8), and hospital-acquired infections (2/8). Among the five patients with postoperative hemorrhage, three experienced early death; two showed a marked postoperative functional decline to KPS ≤ 50, and both died within 3 months without receiving adjuvant treatment. Information regarding post-discharge emergency department attendance was available for 84 patients. During the entire available survival period, 32/84 patients (38.1%) attended the emergency department at least once, accounting for a total of 39 visits. Of these 32 patients, 16 (50.0%) presented with conditions directly related to the neurosurgical procedure, the underlying disease, or ongoing adjuvant treatment. Among disease- or treatment-related emergency department visits, the most common causes were seizures (50.0%) and thrombocytopenia (43.7%) (Table 1).

### 3.2. Radiological Characteristics

Callosal involvement, including tumors involving the splenium, was the most frequent anatomical presentation (30/93, 32.3%), followed by thalamic (18/93, 19.4%), frontal (17/93, 18.3%), temporal (12/93, 12.9%), parietal (7/93, 7.5%), occipital (1/93, 1.1%) brainstem (5/93, 5.4%), and caudate lesions (3/93, 3.2%). 38 lesions (40.9%) were located in the left hemisphere, 30 (32.2%) in the right hemisphere, and 25 (26.9%) showed bilateral involvement in T1-CE. Multicentric disease was observed in 39 patients (41.9%). The median number of additional tumor locations was 1 (IQR, 0–2; range, 0–7). Median contrast-enhancing T1-weighted tumor volume was 15.3 cm^3^ (IQR, 4.1–33.8), and median FLAIR volume was 84.0 cm^3^ (IQR, 31.8–137.0). The median T1-CE-to-FLAIR volume ratio was 0.215 (IQR, 0.076–0.435). A contrast-enhancing butterfly pattern was present in 27 patients (29.0%), while FLAIR signal crossing the midline was observed in 43 patients (46.2%) (Table 2).

### 3.3. Histopathological and Molecular Characteristics

Eighty-seven patients (93.5%) had histologically defined glioblastoma, while 6 (6.5%) had molecularly defined glioblastoma. MGMT promoter methylation status was available in 71 patients, of whom 47 (66.2%) had a methylated promoter and 24 (33.8%) were unmethylated. Among the 55 patients with a quantitative methylation value, the median MGMT methylation level was 16% (IQR, 5–33.5). Median Ki-67 was 25% (IQR, 15–35). TERT promoter alterations were identified in 28 patients (30.1%), while EGFR amplification was identified in one patient (1.1%). All six molecularly defined glioblastomas harbored a TERT promoter alteration (Table 2).

### 3.4. Survival Outcomes

**Figure 2:**
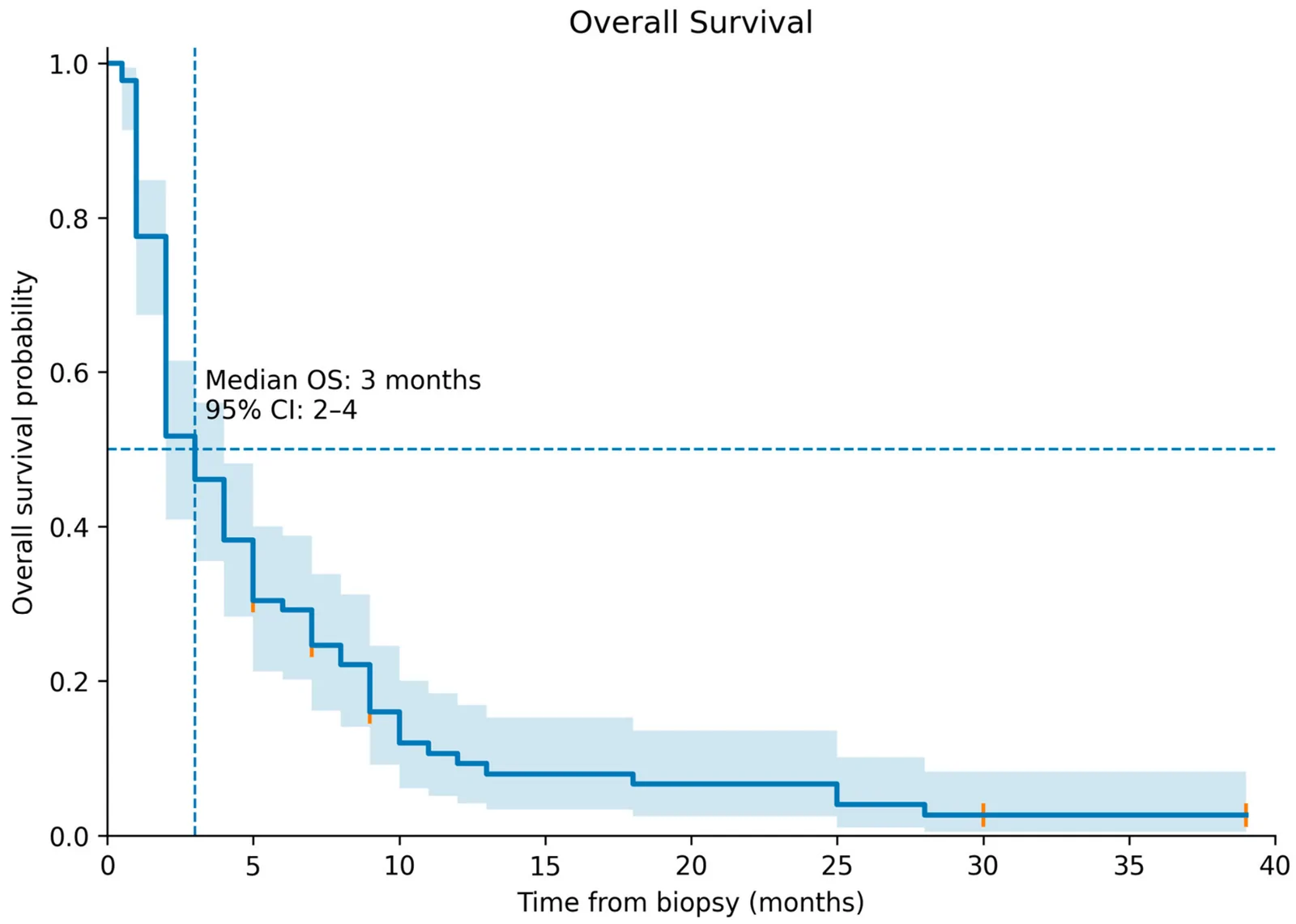
Overall survival of the biopsy-only glioblastoma cohort. Kaplan–Meier estimate of overall survival from the date of biopsy in 89 patients with available follow-up. Shaded areas represent pointwise 95% confidence intervals, and vertical marks indicate censored observations. Dashed lines indicate the median overall survival of 3 months (95% CI, 2–4).

Survival analyses were performed on patients with available follow-up data. Overall survival (OS) was evaluable in 89 of 93 patients (95.7%). During follow-up, 84 deaths were observed and 5 patients were censored. Median OS was 3 months (95% CI, 2–4), and 48 of 89 evaluable patients (53.9%) died within 3 months after biopsy, thereby meeting the definition of early death (Figure 2).

### 3.5. Adjuvant Therapy

The interval between biopsy and initiation of adjuvant treatment had a median duration of 1 month (IQR, 0.75–1.0). Overall, 66 of 93 patients (71.0%) received at least one form of adjuvant treatment, according to a multidisciplinary consensus. Among the 48 patients who died within 3 months, 25 (52.1%) received radiotherapy, chemotherapy, or both. The remaining 23 (47.9%) patients were not considered eligible for any adjuvant treatment. Radiotherapy was administered to 48 patients (51.6% of the overall cohort), with a median total dose of 48 Gy (IQR, 40.4–60.0). Among the 48 patients receiving radiotherapy, 23 (47.9%) received a hypofractionated regimen. Temozolomide-based chemotherapy was administered to 62 patients (66.7%). Chemotherapy had a median duration of 3 months (IQR, 1–6), corresponding to three monthly treatment cycles. 17 patients completed 6 months of treatment (18.3%). Among the 44 patients who received both radiotherapy and temozolomide-based chemotherapy, complete dosing data were available for 43. In these patients, the median cumulative temozolomide dose was 9200 mg (IQR, 5900–13,075), while the median relative dose intensity was 100.0% (IQR, 80.6–100.0). Among the 44 patients receiving both radiotherapy and temozolomide, 24 (54.5%) received concomitant chemoradiotherapy with an initial temozolomide dose of 140 mg. All patients received dexamethasone at a dose of 16 mg/day starting on the day of the biopsy, with gradual tapering over the following weeks (Table 3).

### 3.6. Univariable Analyses of Early Death

Among clinical variables, preoperative KPS was associated with early death. Although median KPS was 80 in both groups, its distribution was shifted toward lower values among patients who died within 3 months (IQR, 60–80 vs. 70–90; Mann–Whitney U *p* = 0.048). Early death occurred in 13 of 16 patients (81.3%) with KPS ≤ 60, compared with 35 of 73 patients (47.9%) with KPS > 60 (odds ratio [OR], 4.70; 95% CI, 1.24–17.91; Fisher exact *p* = 0.025). Patients experiencing early death were descriptively older than those without early death (median, 72 years [IQR, 62.8–77.3] vs 68 years [IQR, 60–77]), although this difference was not significant (*p* = 0.354).

MGMT promoter methylation was associated with lower early mortality. Early death occurred in 17 of 43 patients (39.5%) with methylated MGMT and in 17 of 24 patients (70.8%) with unmethylated MGMT (OR for methylated MGMT, 0.27; 95% CI, 0.09–0.79; *p* = 0.021). Quantitative Ki-67 levels were not significantly different between patients with and without early death (median, 25.0% vs 21.5%; *p* = 0.840). No significant difference was observed between molecularly and histologically defined glioblastoma (early death, 66.7% vs 53.0%; *p* = 0.683). Higher frailty also showed an association with early mortality. Patients with mFI-5 ≥ 2 experienced early death more frequently than those with mFI-5 < 2 (71.4% vs 45.9%; OR, 2.95; 95% CI, 1.13–7.71; Fisher exact *p* = 0.039), although this association did not remain significant after FDR correction (*q* = 0.130). When analyzed as a continuous variable, mFI-5 showed only a non-significant trend toward increased odds of early death (OR per one-point increase, 1.52; 95% CI, 0.93–2.50; *p* = 0.096).

**Figure 3:**
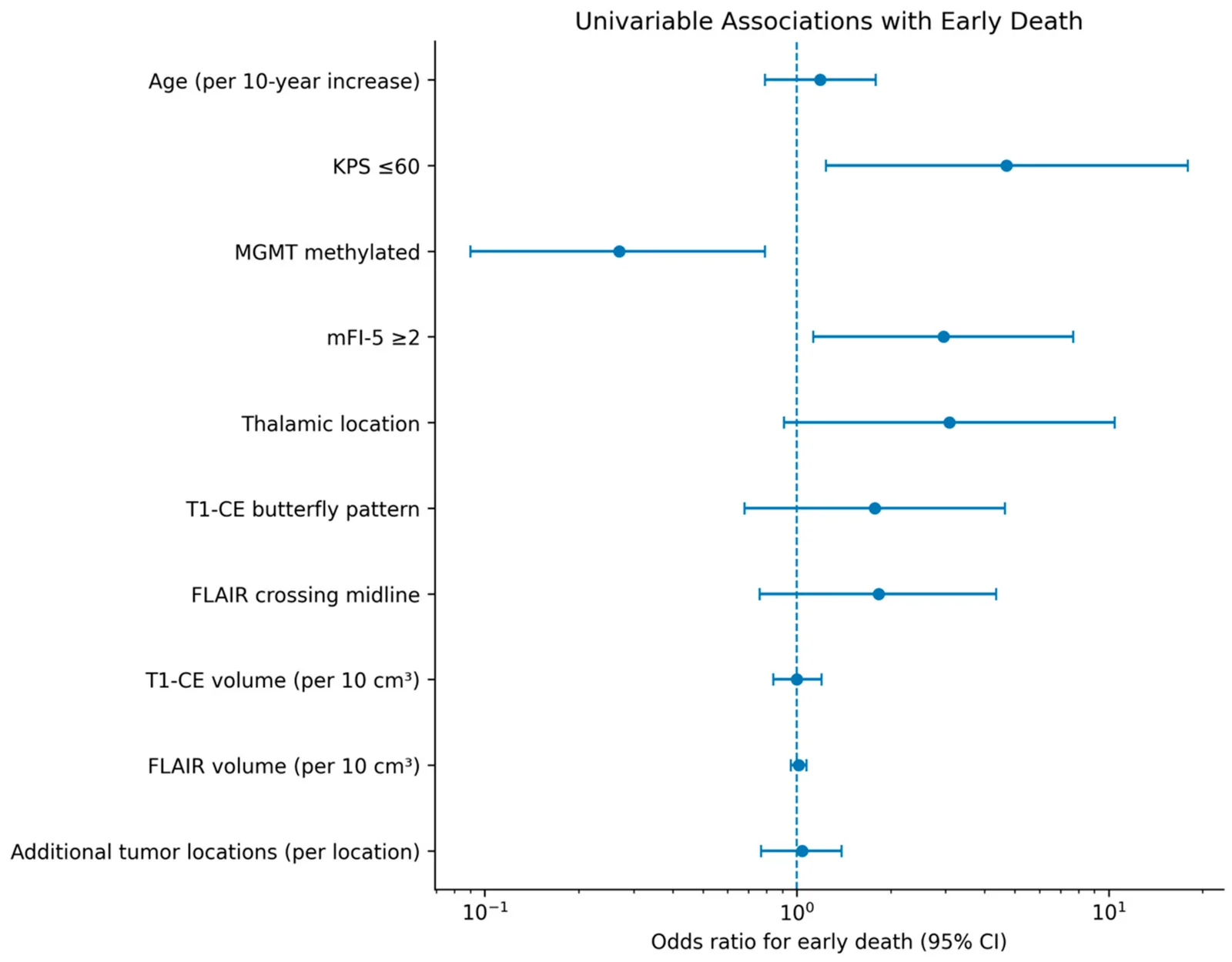
Univariable associations between baseline characteristics and early death. Forest plot showing odds ratios (ORs) and 95% confidence intervals (CIs) for death within 3 months after biopsy. Preoperative KPS ≤ 60 and mFI-5 ≥ 2 were associated with increased odds of early death, whereas MGMT promoter methylation was associated with reduced odds. Age was not significantly associated with early mortality, while thalamic location showed a non-significant trend toward increased odds of early death. Other conventional radiological features, including tumor volumes, butterfly pattern, FLAIR crossing the midline, and number of additional tumor locations, showed no significant individual associations. The vertical dashed line indicates an OR of 1. Continuous variables are expressed according to the increments specified in the figure.

Individual radiological characteristics were not significantly associated with early death. Early-death rates were 57.9% versus 52.2% according to multicentricity (*p* = 0.663), 64.0% versus 50.0% according to the presence of a T1-ce butterfly pattern (OR, 1.78; 95% CI, 0.68–4.65; *p* = 0.340), and 62.5% versus 47.7% according to FLAIR crossing of the midline (OR, 1.83; 95% CI, 0.76–4.36; *p* = 0.195). Thalamic location showed an unfavorable but non-significant association with early death (OR, 3.0; *p* = 0.096). Contrast-enhancing T1 volume (median, 16.0 vs 10.9 cm^3^; *p* = 0.292), FLAIR volume (85.3 vs 84.1 cm^3^; *p* = 0.477), T1-ce-to-FLAIR ratio (*p* = 0.595), and number of tumor locations (*p* = 0.912) were not significantly different between outcome groups. Univariable logistic regression likewise showed no association between early death and T1-ce volume (OR per 10-cm^3^ increase, 1.00; 95% CI, 0.84–1.20; *p* = 0.970), FLAIR volume (OR per 10-cm^3^ increase, 1.013; 95% CI, 0.957–1.073; *p* = 0.659), or number of additional locations (OR per additional location, 1.04; 95% CI, 0.77–1.39; *p* = 0.809) (Figure 3, Table 4). None of these associations remained statistically significant after Benjamini–Hochberg correction for multiple comparisons (all *q* > 0.05), and they should therefore be interpreted as nominal exploratory signals.

### 3.7. Two-Factor Phenotype

In a restricted exploratory analysis of predefined two-factor phenotypes, the combination of preoperative KPS ≤ 60 and FLAIR signal crossing the midline identified a small subgroup at particularly high risk of early death. Nine of 10 patients presenting with both characteristics died within 3 months (90.0%; Wilson 95% CI, 59.6–98.2%), compared with approximately 50% of the remaining evaluable patients (OR, 9.00; 95% CI, approximately 1.08–75.0; Fisher exact *p* = 0.020). This phenotype had high specificity (97.4%) but low sensitivity (19.6%) for early death. Given the small size of this subgroup (*n* = 10) and the wide confidence interval, this estimate should be interpreted cautiously. The association did not remain statistically significant after false-discovery-rate correction and should therefore be interpreted as hypothesis-generating rather than as a validated treatment-selection criterion (Table 4).

### 3.8. Multivariable Analyses

**Figure 4:**
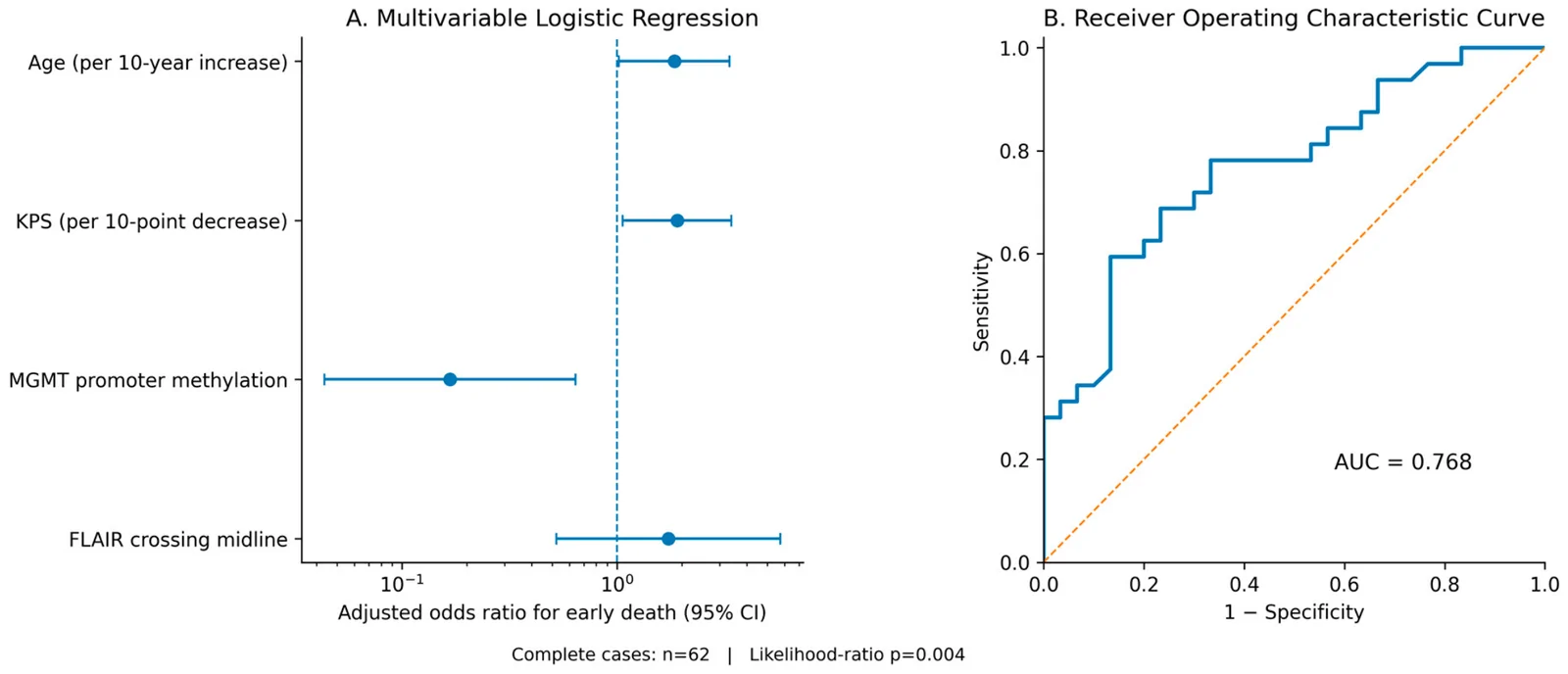
Exploratory multivariable analysis of early death and model discrimination. (**A**) Forest plot showing adjusted odds ratios (aORs) and 95% confidence intervals (CIs) from the multivariable binary logistic regression model including age, preoperative KPS, MGMT promoter methylation status, and FLAIR signal crossing the midline. (**B**) Receiver operating characteristic (ROC) curve showing the apparent discriminative ability of the multivariable model for early death. The analysis included 62 complete cases. The overall model was statistically significant (likelihood-ratio *p* = 0.004) and showed an area under the ROC curve (AUC) of 0.768. The AUC represents apparent discrimination within the study cohort and was not internally or externally validated.

An exploratory multivariable binary logistic regression model was fitted in 62 complete cases, including 32 early deaths. The model incorporated age, preoperative KPS, MGMT promoter methylation, and FLAIR signal crossing the midline as four clinically prespecified covariates representing distinct prognostic domains. Each 10-year increase in age was associated with higher odds of early death (adjusted OR [aOR], 1.84; 95% CI, 1.02–3.33; *p* = 0.043), as was each 10-point decrease in KPS (aOR, 1.90; 95% CI, 1.06–3.40; *p* = 0.031). MGMT promoter methylation was independently associated with lower early mortality (aOR, 0.17; 95% CI, 0.04–0.64; *p* = 0.009). FLAIR crossing the midline showed an unfavorable but non-significant association (aOR, 1.73; 95% CI, 0.52–5.74; *p* = 0.371). The overall model was statistically significant (likelihood-ratio *p* = 0.004) and showed moderate apparent discrimination (area under the receiver-operating-characteristic curve, 0.768). Among the 89 patients with available survival data, missingness affecting the multivariable model was limited to MGMT status (*n* = 22) and FLAIR crossing-midline status (*n* = 5), resulting in 62 complete cases. Patients included and excluded from the complete-case analysis did not significantly differ in age, preoperative KPS, mFI-5, early mortality, calendar era, or major radiological characteristics. In a sensitivity analysis excluding MGMT and including 84 patients, preoperative KPS remained associated with early mortality (OR 1.53 per 10-point decrease, 95% CI 1.01–2.30; *p* = 0.045) and the addition of mFI-5 to the multivariable model did not provide significant incremental prognostic information (aOR per one-point increase, 1.62; 95% CI, 0.68–3.88; *p* = 0.279), with virtually unchanged model discrimination (AUC, 0.769 vs 0.768). Given the complete-case sample size, absence of external validation, and exploratory design, the model was not considered suitable for individual prognostic prediction or clinical treatment selection (Figure 4, Table 4).

## 4. Discussion

In this study, we investigated potential predictors of early mortality, defined as death within three months of surgery, in patients with unresectable glioblastoma undergoing biopsy alone. Preoperative KPS and MGMT promoter methylation status showed the strongest associations with early mortality. However, neither remained statistically significant after FDR correction, limiting the strength of these exploratory findings. Although age was not significantly associated with early death in the univariable analysis, it was associated with early mortality after adjustment in the exploratory multivariable model.

Radiological parameters did not show clear evidence of association with early mortality in our cohort, either in univariable or multivariable analyses, although the sample size and resulting statistical precision limit definitive conclusions regarding their prognostic value. However, an exploratory analysis identified a potential association between FLAIR signal crossing the midline, a preoperative KPS ≤ 60, and death within three months after biopsy.

Our findings are broadly consistent with previous biopsy-only glioblastoma series, while also showing some relevant differences. Edriss et al., using the same three-month survival endpoint in an elderly biopsy-only population, identified poor performance status, larger tumor volume, and central tumor location as independent predictors of early mortality [9]. While our results confirm the central prognostic role of functional status, tumor volume and other conventional radiological features did not show consistent associations with early death. Conversely, MGMT promoter methylation and, after multivariable adjustment, age appeared to provide additional prognostic information in our cohort. This may suggest that short-term outcome in biopsy-only glioblastoma cannot be explained by anatomical tumor burden alone.

Our cohort also showed a particularly unfavorable clinical course compared with previous biopsy-only series. Gonçalves et al. reported that 77.8% of patients survived beyond three months, with a median OS of 6 months, while Löber-Handwerker et al. reported a median OS of 5.5 months [8,9]. In our cohort, median OS was 3 months and 53.9% of evaluable patients died within three months. This difference may partly reflect the particularly unfavorable baseline profile of our biopsy-only population: 58.1% had a deep tumor location, 62.4% had multicentric disease, 29.0% showed a contrast-enhancing butterfly pattern, 17.2% had a preoperative KPS ≤ 60, and 32.3% had an mFI-5 ≥ 2. Differences in calendar era, referral patterns, patient selection, and treatment strategies across retrospective series may further contribute to the observed survival variability and limit direct comparisons between cohorts.

One of the most relevant findings from the descriptive analysis was the high proportion of patients experiencing early death who never initiated adjuvant treatment (47.9%, 23/48). Our study specifically sought to identify factors that may allow earlier recognition of this particularly vulnerable subgroup. Such information could support timely and individualized treatment planning and provide patients, caregivers, and treating physicians with additional prognostic information to facilitate shared decision-making and care planning.

Importantly, our findings should not be interpreted as questioning the diagnostic role of biopsy in patients with MRI findings suggestive of glioblastoma, even in the presence of clinical or radiological features associated with a poor prognosis. MRI plays an essential role in the diagnostic work-up of suspected glioblastoma but cannot currently replace histopathological confirmation. In a prospective series of adults with MRI findings suggestive of brainstem glioma, only 61% of lesions were ultimately confirmed as gliomas following biopsy [18]. Moreover, conventional and advanced MRI techniques have shown imperfect diagnostic performance in differentiating glioblastoma from other intracranial lesions, including solitary brain metastases, primary central nervous system lymphoma, and brain abscesses [19,20,21]. Therefore, tissue sampling by biopsy or surgical resection remains essential for establishing an integrated histomolecular diagnosis and guiding subsequent oncological treatment, including in patients presenting with unfavorable baseline clinical characteristics [6].

The high rate of early mortality observed in our cohort should also be interpreted in the context of the limited ability of these patients to undergo standard oncological treatment. Overall, 23 patients (24.7%) were not considered eligible for any adjuvant therapy, while only 17 (18.3%) completed the full Stupp regimen. Postoperative complications may further contribute to this clinical vulnerability: among the five patients experiencing postoperative hemorrhage, the two with a marked functional decline to KPS ≤ 50 both experienced early death and did not subsequently receive adjuvant treatment. These findings reflect the narrow therapeutic window that characterizes a substantial proportion of patients with biopsy-only glioblastoma. The management of biopsy-only glioblastoma is further complicated by the lack of treatment strategies specifically validated for this heterogeneous population. Although current guidelines provide general recommendations based on age, performance status, and MGMT promoter methylation, the optimal choice and intensity of adjuvant treatment remain poorly defined, particularly in frail patients with unresectable disease. Consequently, treatment decisions are often individualized, resulting in substantial variability in radiotherapy schedules, chemotherapy regimens, dose intensity, and treatment duration across clinical practice and published series. This heterogeneity complicates real-world comparisons and limits the ability to determine which therapeutic approaches may provide the greatest benefit in specific subgroups.

In this setting, overall survival may not fully capture the clinical benefit of treatment. Harlay et al. recently proposed duration of functional autonomy as an additional clinically relevant outcome in biopsy-only glioblastoma [5]. Our findings support a similar patient-centered perspective. Early prognostic stratification may help distinguish patients who are likely to undergo and potentially benefit from standard treatment from those at high risk of rapid clinical deterioration. This should not be interpreted as a rationale for treatment exclusion, but rather as an opportunity to tailor therapeutic intensity, provide realistic prognostic information, and integrate supportive and palliative care at an appropriate stage of the disease course.

The main limitations of our study reside in its retrospective, single-center design and the relatively limited sample size may restrict the generalizability of our findings. Missing data for some molecular and clinical variables further reduced the number of patients available for multivariable analyses. Moreover, the association between low KPS and FLAIR crossing should be considered hypothesis-generating rather than predictive and requires validation in independent cohorts. The single-center design may limit the generalizability of our findings, which require validation in larger, independent multicenter cohorts. Future multicenter studies should determine whether the integration of clinical, molecular, and imaging features can improve early prognostic stratification in biopsy-only glioblastoma and support treatment selection without compromising access to potentially beneficial therapies.

## 5. Conclusions

In this biopsy-only glioblastoma cohort, preoperative functional status, MGMT promoter methylation, age, and selected radiological features showed associations with short-term mortality that warrant validation in larger, independent multicenter cohorts. The high rate of early mortality and the proportion of patients who never initiated adjuvant treatment highlight the clinical vulnerability and heterogeneity of this population. These findings may contribute to future prognostic stratification but do not establish a validated prognostic tool or a treatment-selection rule.

## Figures and Tables

**Table 1:** Baseline clinical characteristics of the biopsy-only glioblastoma cohort. Data are presented as median (interquartile range [IQR]), *n* (%), or *n*/*N* (%), as appropriate. Complication categories were not mutually exclusive. Denominators are reported when data were not available for the entire cohort. KPS, Karnofsky Performance Status; mFI-5, 5-item modified Frailty Index.

Characteristic	Overall Cohort (*N* = 93)
Age at biopsy, years, median (IQR)	70 (61–77)
Sex, *n* (%)	
Female	47 (50.5)
Male	46 (49.5)
Preoperative KPS, median (IQR)	80 (70–80)
Immediate postoperative KPS, median (IQR)	80 (70–80)
mFI-5, median (IQR)	1 (1–2)
Postoperative complications, *n* (%)	8 (8.6)
Intracranial hemorrhage, *n*/*N* (%)	5/8 (62.5)
Hospital-acquired infection, *n*/*N* (%)	2/8 (25.0)
New neurological deficit, *n*/*N* (%)	1/8 (12.5)
Seizure, *n*/*N* (%)	1/8 (12.5)
Post-discharge emergency department attendance, *n*/*N* (%)	32/84 (38.1)
Total emergency department visits, *n*	39
Disease-, procedure-, or treatment-related attendance, *n*/*N* (%)	16/32 (50.0)
Seizure among related attendances, *n*/*N* (%)	8/16 (50.0)
Thrombocytopenia among related attendances, *n*/*N* (%)	7/16 (43.7)

**Table 2:** Radiological, histopathological, and molecular characteristics of the biopsy-only glioblastoma cohort. Data are presented as median (interquartile range [IQR]), n (%), or n/N (%), as appropriate. Percentages refer to the overall cohort unless a different denominator is specified. EGFR, epidermal growth factor receptor; FLAIR, fluid-attenuated inversion recovery; MGMT, O^6^-methylguanine-DNA methyltransferase; mFI-5, 5-item modified Frailty Index; T1-CE, contrast-enhanced T1-weighted imaging; TERT, telomerase reverse transcriptase.

Characteristic	Overall Cohort (*N* = 93)
Radiological Characteristics	
Primary anatomical location, *n* (%)	
Corpus callosum	30 (32.3)
Thalamus	18 (19.4)
Frontal lobe	17 (18.3)
Temporal lobe	12 (12.9)
Parietal lobe	7 (7.5)
Brainstem	5 (5.4)
Caudate nucleus	3 (3.2)
Occipital lobe	1 (1.1)
Laterality, *n* (%)	
Left	38 (40.9)
Right	30 (32.2)
Bilateral (T1-CE)	25 (26.9)
Multicentric disease, *n* (%)	39 (41.9)
Additional tumor locations, range	0–7
Contrast-enhancing T1-weighted tumor volume, cm^3^, median (IQR)	15.3 (4.1–33.8)
FLAIR volume, cm^3^, median (IQR)	84.0 (31.8–137.0)
T1-CE-to-FLAIR volume ratio, median (IQR)	0.215 (0.076–0.435)
Contrast-enhancing butterfly pattern, *n* (%)	27 (29.0)
FLAIR signal crossing the midline, *n* (%)	43 (46.2)
Histopathological and molecular characteristics	
Glioblastoma classification, *n* (%)	
Histologically defined glioblastoma	87 (93.5)
Molecularly defined glioblastoma	6 (6.5)
MGMT promoter methylation status, *n*/*N* (%)	
Methylated	47/71 (66.2)
Unmethylated	24/71 (33.8)
Ki-67 index, *n*/*N* (%), median (IQR)	25/77 (32.5) (15–35)
TERT mutation, *n*/*N* (%)	28/93 (30.1)
EGFR amplification, *n*/*N* (%)	1/93 (1.1)

**Table 3:** Adjuvant treatment administered after biopsy in the overall cohort and in patients who died within three months. Data are presented as median (interquartile range [IQR]), *n* (%), or *n*/*N* (%), as appropriate.

Treatment Characteristic	Overall Cohort (*N* = 93)
Biopsy-to-adjuvant-treatment interval, months, median (IQR)	1.0 (0.75–1.0)
Any adjuvant treatment, *n* (%)	66 (71.0)
Radiotherapy, *n* (%)	48 (51.6)
Total radiotherapy dose, Gy, median (IQR)	48.0 (40.4–60.0)
Temozolomide-based chemotherapy, *n* (%)	62 (66.7)
Chemotherapy duration, months, median (IQR)	3 (1–6)
Completed 6 months of treatment, *n* (%)	17 (18.3)
Combined radiotherapy and chemotherapy, *n* (%)	44 (47.3)
Cumulative temozolomide dose, mg, median (IQR)	9200 (5900–13,075); *n* = 43
Relative dose intensity, %, median (IQR)	100.0 (80.6–100.0); *n* = 43
Patients with early death (*n* = 48)	
Received any adjuvant treatment, *n*/*N* (%)	25/48 (52.1)
Not eligible for adjuvant treatment, *n*/*N* (%)	23/48 (47.9)
Dexamethasone treatment from the day of biopsy, *n* (%)	93 (100)
Initial dexamethasone dose, mg/day	16

**Table 4:** Univariable, two-factor phenotype, and exploratory multivariable analyses of early death within 3 months after biopsy. Odds ratios (ORs) and 95% confidence intervals (CIs) are reported for associations with early death. False-discovery-rate (FDR) q-values were calculated using the Benjamini–Hochberg procedure across exploratory baseline comparisons. The predefined two-factor phenotype combining preoperative KPS ≤ 60 and FLAIR signal crossing the midline was associated with a 90.0% early-death rate (9/10; Wilson 95% CI, 59.6–98.2%). The multivariable logistic regression included age, preoperative KPS, MGMT promoter methylation status, and FLAIR crossing the midline and was performed on 62 complete cases. Adjusted odds ratios (aORs) are reported for the multivariable model. Bold values indicate statistical significance at *p* < 0.05. AUC, area under the receiver operating characteristic curve; CI, confidence interval; FDR, false discovery rate; KPS, Karnofsky Performance Status; MGMT, O6-methylguanine-DNA methyltransferase; mFI-5, 5-item modified Frailty Index; OR, odds ratio.

Variable/Comparison	OR (95% CI)	*p*-Value	FDR *q*-Value
**Univariable analysis**			
Age, per 10-year increase	1.19 (0.79–1.79)	0.398	0.596
Preoperative KPS ≤ 60	4.70 (1.24–17.91)	**0.025**	0.125
MGMT promoter methylated	0.27 (0.09–0.79)	**0.021**	0.125
mFI-5 ≥ 2	2.95 (1.13–7.71)	**0.039**	0.130
Thalamic location	3.08 (0.91–10.46)	0.095	0.238
T1-CE butterfly pattern	1.78 (0.68–4.65)	0.277	0.462
FLAIR crossing midline	1.83 (0.76–4.36)	0.184	0.368
T1-CE volume, per 10 cm^3^ increase	1.00 (0.84–1.20)	0.997	0.997
FLAIR volume, per 10 cm^3^ increase	1.013 (0.957–1.073)	0.652	0.815
Additional tumor locations, per location	1.04 (0.77–1.39)	0.820	0.911
**Exploratory two-factor phenotype**			
KPS ≤ 60 + FLAIR crossing midline	9.00 (1.08–75.0)	**0.020**	0.170
**Exploratory multivariable analysis**	**aOR (95% CI)**		
Age, per 10-year increase	1.84 (1.02–3.33)	**0.043**	—
Preoperative KPS, per 10-point decrease	1.90 (1.06–3.40)	**0.031**	—
MGMT promoter methylated	0.17 (0.04–0.64)	**0.009**	—
FLAIR crossing midline	1.73 (0.52–5.74)	0.371	—

## Data Availability

The raw data supporting the conclusions of this article will be made available by the authors on request.

## Abbreviations

The following abbreviations are used in this manuscript:

GBM	Glioblastoma
OS	Overall Survival
MGMT	O^6^-methylguanine-DNA methyltransferase
KPS	Karnofsky Performance Status
IDH	Isocitrate Dehydrogenase
CI	Confidence Interval
TMZ	Temozolomide
IQR	Interquartile Range
mFI-5	5-item modified Frailty Index
